# Multinational Association of Supportive Care in Cancer (MASCC) expert opinion/guidance on the use of clinically assisted nutrition in patients with advanced cancer

**DOI:** 10.1007/s00520-021-06613-y

**Published:** 2021-10-19

**Authors:** Bryony Alderman, Lindsey Allan, Koji Amano, Carole Bouleuc, Mellar Davis, Stephanie Lister-Flynn, Sandip Mukhopadhyay, Andrew Davies

**Affiliations:** 1 Royal Surrey County Hospital, Guildford, UK; 2 Department of Palliative Medicine, National Cancer Center Hospital, Tokyo, Japan; 3 Department of Supportive and Palliative Care, Institut Curie, Paris, France; 4 Palliative Care Department, Geisinger Medical System, PA Danville, USA; 5 St Catherine’s Hospice, Crawley, UK; 6 Department of Pharmacology, Burdwan Medical College, Burdwan, India; 7 School of Medicine, Trinity College Dublin, Dublin, Ireland; 8 Education & Research Centre, Our Lady’s Hospice Dublin, Harold’s Cross, Dublin, Ireland; 9 School of Medicine, University College Dublin, Dublin, Ireland

**Keywords:** Clinically assisted nutrition, Neoplasms, Palliative care, Practice guideline, Advanced cancer

## Abstract

**Purpose:**

The pro vision of clinically assisted nutrition (CAN) in patients with advanced cancer is controversial, and there is a paucity of specific guidance, and so a diversity in clinical practice. Consequently, the Palliative Care Study Group of the Multinational Association of Supportive Care in Cancer (MASCC) formed a Subgroup to develop evidence-based guidance on the use CAN in patients with advanced cancer.

**Methods:**

This guidance was developed in accordance with the MASCC Guidelines Policy. A search strategy for Medline was developed, and the Cochrane Database of Systematic Reviews and the Cochrane Central Register of Controlled Trials were explored for relevant reviews/trials respectively. The outcomes of the review were categorised by the level of evidence, and a “category of guideline” based on the level of evidence (i.e. “recommendation”, “suggestion”, or “no guideline possible”).

**Results:**

The Subgroup produced 11 suggestions, and 1 recommendation (due to the paucity of evidence). These outcomes relate to assessment of patients, indications for CAN, contraindications for CAN, procedures for initiating CAN, and re-assessment of patients.

**Conclusions:**

This guidance provides a framework for the use of CAN in advanced cancer, although every patient needs individualised management.

**Supplementary Information:**

The online version contains supplementary material available at 10.1007/s00520-021-06613-y.

## Introduction

The decision to initiate (or withdraw) clinically assisted nutrition (CAN) in patients with advanced cancer is a common clinical scenario. In some cases, the decision appears relatively straightforward, whilst in many cases, the decision depends on a subjective assessment of the potential benefits versus the potential risks. Research suggests that, especially at the end of life, the use of CAN varies enormously (3–53%) [1], and that patients and their families often have very positive views about CAN, whilst healthcare professionals often have disparate views about CAN [2–4].

On the basis of the above, the Palliative Care Study Group of the Multinational Association of Supportive Care in Cancer (MASCC) formed a Subgroup to develop evidence-based guidance on the use of CAN in patients with advanced cancer. This paper gives an overview of CAN in patients with advanced cancer, the methodology involved in developing the outcomes, and the evidence to support the outcomes (and the grading of the evidence).

At the time the Subgroup started the project, there were no up-to-date guidelines on the use of CAN in patients with advanced cancer, although there are older guidelines relating to this cohort of patients [5, 6], and there are newer guidelines relating to cancer patients in general (which address this cohort of patients to a minor extent) [7, 8]. Our guidance complements the latter guidelines, and is aimed at the core multidisciplinary team involved in the care of patients with advanced cancer.

## Background

### Definitions

For the purposes of this guidance, CAN refers to all forms of tube-feeding (e.g. via nasogastric tube, percutaneous endoscopic gastrostomy (PEG), or parenteral nutrition (PN). It does not cover oral feeding, by cup, spoon, or any other method of delivering food or nutritional supplements into a patient’s mouth [9]. Synonymous terms within the medical literature include “medically-assisted nutrition” [10], “artificial nutrition” [5], “artificial feeding” [11], and “hyperalimentation” (specifically for parenteral nutrition) [12]. The term “medical nutrition therapy” includes the use of oral nutritional supplements as well as “tube feeding” [13].

Other definitions used in this guidance include “advanced cancer” (i.e. “cancer that is unlikely to be cured or controlled with treatment. The cancer may have spread from where it first started, to nearby tissue, lymph nodes, or distant parts of the body. Treatment may be given to help shrink the tumour, slow the growth of cancer cells, or relieve symptoms”) [14], “end-of-life” (i.e. the last year of life) [15], and “terminal phase” (i.e. the last days to weeks of life) [16]. It should be noted that patients with advanced cancer may not be at the end-of-life (as defined), and that prognostication remains exceptionally challenging (especially when the prognosis is of the order of months to years rather than days to weeks) [17]. Thus, the trajectory of the illness may change (i.e. accelerate or decelerate), and/or acute events may intervene (i.e. cancer-related or separate condition).

### Nutritional requirements

**Fig. 1:**
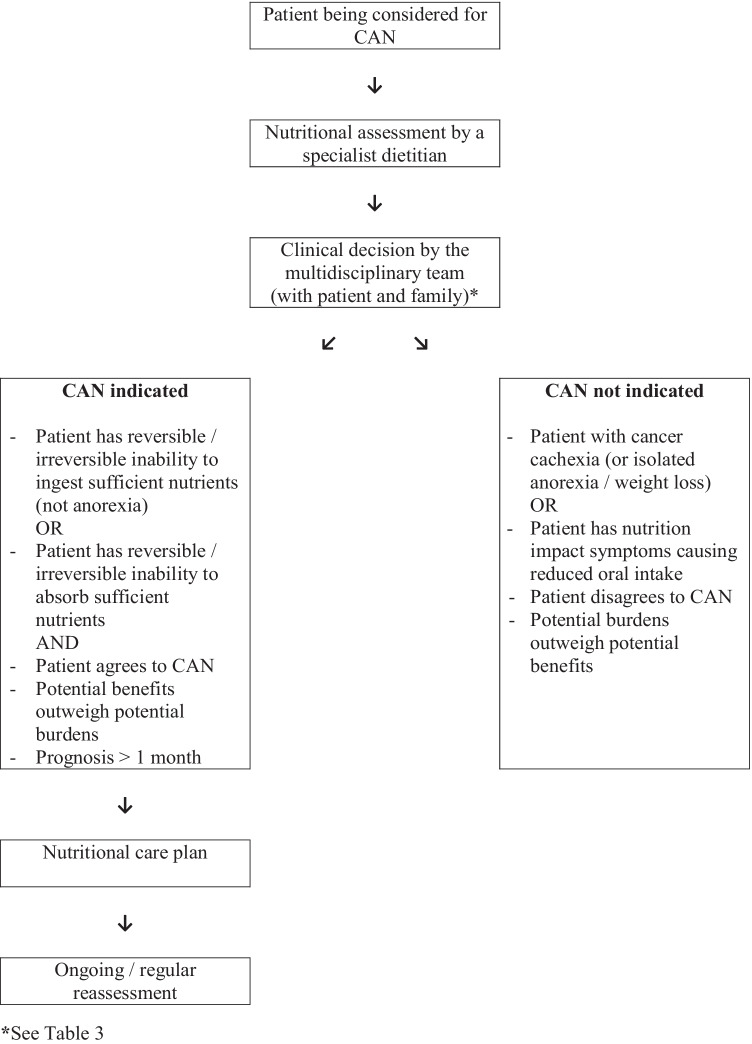
Decision algorithm for CAN in patients with advanced cancer.

The National Institute for Health and Care Excellence/NICE (United Kingdom) recommend a “total intake” for all adults that includes [18] (a) 25–35 kcal/kg/day total energy; (b) 0.8–1.5 g protein (0.13–0.24 g nitrogen)/kg/day; (c) 30–35 ml fluid/kg (allowing for excessive losses, and other sources of fluids); and (d) adequate electrolytes, minerals, micronutrients, and fibre. Other guidelines recommend similar amounts of nutrients for cancer patients [7, 19] Fig. 1.

### Malnutrition

Malnutrition (also known as undernutrition) has been defined as “a state resulting from lack of intake or uptake of nutrition that leads to altered body composition (decreased fat free mass) and body cell mass leading to diminished physical and mental function and impaired clinical outcome from disease” [13]. Malnutrition can result from starvation, disease (gastrointestinal disease, acute injury, acute systemic disease with inflammation, chronic systemic disease with/without inflammation), normal ageing, or a combination of these factors [13, 20]. Consensus diagnostic criteria for malnutrition include the presence of one so-called phenotypic criterion (i.e. weight loss, reduced body mass index, reduced muscle mass), and one so-called etiologic criterion (i.e. reduced food intake or assimilation, disease burden/inflammation) [20].

Malnutrition remains a major cause of mortality worldwide, and it has been estimated that malnutrition is the direct cause of death in 10–20% cancer patients [21]. Data on the Irish Republican Army (IRA) hunger strikers suggests that on average, an otherwise healthy young adult male can survive for 61 days without food [22]: the minimum recorded survival was 46 days, whilst the maximum recorded survival was 73 days [23]. However, survival would be expected to be “considerably reduced” in patients with an underlying malignancy [24]. Importantly, malnutrition also results in significant morbidity. Every system within the body is affected, resulting in physical (e.g. muscle weakness), cognitive (e.g. impaired memory), and psychological problems (e.g. depression), with associated impact on quality of life, and the ability to undertake activities of daily living [24, 25]

.

### Nutritional problems in cancer patients

#### Anorexia

Anorexia (loss of appetite) is a common symptom in patients with advanced cancer (30–92%) [26, 27], and is especially prevalent in patients at the end-of-life and in the terminal phase. Anorexia often leads to weight loss, although this is not an inevitable consequence. Anorexia may be related to a number of potentially reversible factors (e.g. “nutrition impact symptoms” — see below), and may be amenable to specific interventions (e.g. corticosteroids, progestogens) [28], as well as use of supportive measures (i.e. dietary advice, use of oral nutritional supplements). CAN should never be initiated solely on the basis of the development of anorexia (causing reduced oral intake).

#### Weight loss

Weight loss is also a common problem in patients with advanced cancer (33–93%) [26, 27], and is especially prevalent in patients at the end-of-life and in the terminal phase. As discussed, anorexia often leads to weight loss, but paradoxically malnutrition often leads to anorexia [24]. Weight loss may also be related to a number of potentially reversible factors (e.g. “nutrition impact symptoms” — see below), and again may be amenable to specific interventions (e.g. corticosteroids, progestogens) [28], as well as use of supportive measures (i.e. dietary advice, use of oral nutritional supplements). CAN should never be initiated solely on the basis of the development of weight loss.

#### Nutrition impact symptoms

Nutrition impact symptoms (NIS) are a range of symptoms/problems that interfere with the patient’s appetite, their ability to ingest food, or their ability to digest food [29]. Examples of NIS include dry mouth, taste disturbance, oral discomfort, dental/denture problems, difficulty swallowing, nausea, vomiting, early satiety, constipation, and certain systemic symptoms/problems (e.g. fatigue, low mood).

NIS form part of certain assessment tools (e.g. Patient-Generated Subjective Global Assessment / PG-SGA) [30], and specific a checklist has been developed for patients with advanced cancer [29]. However, none of these tools include a complete list of NIS, and most cancer-related/cancer treatment-related symptoms have the potential to interfere with patient nutrition (either directly, or indirectly).

#### Malnutrition

Malnutrition is common in patients with cancer (20–70%), with the prevalence dependent on the cancer type, the cancer stage, and the age of the patient [21]. Thus, malnutrition is more common in patients with head and neck, lung, and gastrointestinal cancers: malnutrition is also more common in patients with advanced disease (cf. early cancer), and more common in older patients (cf. younger patients) [21].

#### Cancer cachexia

Cancer cachexia is a distinct type of disease-associated malnutrition [13], which is common in patients with advanced cancer (~ 50%) [28]. It is the result of a variable combination of reduced food intake and abnormal metabolism [31]. The metabolic alterations are highly complex (and not completely understood), but prominent features include systemic inflammation, and increased catabolism (or decreased anabolism) [32]. Importantly, for the reasons outlined, medical nutritional therapies (including CAN) per se are ineffective in managing cancer cachexia [28].

International consensus diagnostic criteria for cancer cachexia are (a) weight loss > 5% over 6 months (in absence of simple starvation); or (b) Body Mass Index (BMI) < 20 and any degree of weight loss > 2%; or (c) appendicular skeletal muscle index consistent with sarcopenia (males < 7.26 kg/m^2^; females < 5.45 kg/m^2^) and any degree of weight loss > 2% [31]. Of note, the wasting process in cancer cachexia is somewhat different from the wasting process in simple starvation: in the former, the predominant factor is skeletal muscle loss (with or without loss of adipose tissue), whilst in the latter, the predominant factor is loss of adipose tissue (with preservation of skeletal muscle).

### Nutritional therapies

Nutritional therapies include oral nutritional supplements, enteral tube feeding (also known as enteral nutrition), and parenteral nutrition [13]. Enteral tube feeding involves the delivery of nutrients via a tube (e.g. nasogastric/NG; nasojejunal/NJ), or via a stoma (e.g. percutaneous endoscopic gastrostomy/PEG; percutaneous jejunostomy/PEJ). Enteral tube feeding may be total (TEN), or supplemental to oral intake of food. Parenteral nutrition (PN) involves delivery of nutrients through a peripheral venous line or a central venous line. Parenteral nutrition may also be total (TPN), or supplemental to oral intake of food (SPN).

CAN is considered a medical treatment, and recent European Society for Clinical Nutrition and Metabolism (ESPEN) guidelines highlight the ethical principles regarding the provision/omission of CAN (Table 1) [8]. This guideline is based on universal ethical principles (i.e. autonomy, beneficence, non-maleficence, justice), but readers are encouraged to check their own national guidance on the provision/omission of CAN/medical treatments.

**Table.1:** Ethical considerations relating to provision of clinically assisted nutrition in patients with advanced cancer [8]

The physician / multidisciplinary team has the ultimate responsibility for making the decision on clinically assisted nutrition
Clinically assisted nutrition should be considered if the potential benefits outweigh the potential burdens (and vice versa)
Clinically assisted nutrition should be considered if it is unclear whether the potential benefits outweigh the potential burdens (i.e. give a trial of clinically assisted nutrition)
The patient does not have the right to demand clinically assisted nutrition
The patient does have the right to refuse clinically assisted nutrition (if the patient has capacity / competence)
A valid advance directive to refuse treatment must be followed (if the patient does not have capacity / competence)
The family do not have the right to demand clinically assisted nutrition

## Methods

The aim of the Subgroup was to develop comprehensive, clinically relevant, evidence-based guidance on the provision of CAN in patients with advanced cancer. Thus, it was agreed that the guidance could include ones supported by “high” levels of evidence (e.g. systematic reviews), as well as ones supported by “low” levels of evidence (e.g. expert opinion), if the topic were deemed to be clinically relevant.

The guidance was developed in accordance with the MASCC Guidelines Policy [33]. The Subgroup adopted the National Cancer Institute (NCI) definition of advanced cancer (see above) [14], and data was included from studies involving cancer patients still receiving anti-cancer treatment, and also cancer patients only receiving palliative care (or both modalities).

A search strategy for Medline was developed (Appendix 1), and the Cochrane Database of Systematic Reviews and the Cochrane Central Register of Controlled Trials (CENTRAL) were explored for relevant reviews/trials respectively [34, 35]. The review of the published literature was restricted to papers written in English, and to papers relating to adult (≥ 19 years) humans.

All abstracts identified by the search of Medline (1946 to 10th July 2020) were downloaded into a reference management software package. These abstracts were independently assessed for relevance by the two main authors (BA, AD), and if one author deemed the abstract relevant, then the full text of the article was obtained. These articles were independently assessed for inclusion by the two main authors. All of the authors were involved in assessing the randomised controlled trials in the CENTRAL, and the two main authors were involved in assessing the systematic reviews in the Cochrane Database of Systematic Reviews.

Whenever possible, the guidance was based on data from patients with advanced cancer. However, when no data was available, or only poor-quality data was available, then data from other populations was extrapolated (if deemed appropriate). The outcomes of the review were characterised by a level of evidence (i.e. I, II, III, IV, or V), and a “category of guideline” based on the level of evidence (i.e. “recommendation”, “suggestion”, or “no guideline possible”) (Appendix 2) [33]. The outcomes were independently characterised by the two main authors (BA, AD), and a consensus reached in the case of any disagreement. All of the authors agreed with the outcomes/characterisations of outcomes.

## Results

The searches were last undertaken on 13th July 2020. The Medline search identified 1513 references, and 110 full text articles were retrieved (and reviewed). The search of the Cochrane Central Register of Controlled Trials (29th July 2020) identified 1368 references, and 11 more full-text articles were formally examined. Similarly, the search of the Cochrane Database of Systematic Reviews (29th July 2020) identified 39 references, and 4 reviews were formally examined. Reference lists of the retrieved articles/reviews were also checked for additional sources of information (not identified in the original searches).

The Subgroup were only able to formulate 11 suggestions, and 1 recommendation (due to the paucity of evidence).

### Outcomes of review

The suggestions/recommendation of the Subgroup are summarised in Table 2 (with the levels of evidence, and the categories of guideline).

1. All patients with advanced cancer should have regular nutritional assessments [Level of evidence—V; category of guideline—suggestion].
  All patients with advanced cancer should be regularly assessed. Initial assessment (screening) involves evaluation of current food intake, and recent weight change (loss), together with measurement of BMI [7]: subsequent assessment depends on the individual clinical situation (measurement of body composition, e.g. muscle mass; measurement of inflammatory biomarkers, e.g. C-reactive protein). A number of validated nutritional screening tools are available to facilitate screening (e.g. Nutrition Risk Screening 2002/NRS-2002, Malnutrition Universal Screening Tool/MUST) [7]. All patients with advanced cancer should also be regularly assessed for nutrition impact symptoms (see above).
  Although clearly related, patients require separate assessments for the need for CAN, and the need for clinically assisted hydration [15]. Furthermore, any decision to withhold/withdraw CAN should trigger an urgent review of the need for clinically assisted hydration. The MASCC Palliative Care Study Group are developing analogous guidance on the use of clinically assisted hydration in patients with advanced cancer.
2. Patients with nutritional problems should be reviewed by a specialist dietitian (with/without other members of the nutrition support team) [Level of evidence—V; category of guideline—suggestion].
  Nutritional problems in cancer patients are somewhat different from those in other groups of patients, and so these patients should ideally be reviewed by a specialist dietitian (preferably who has oncology experience), with/without other members of the nutrition support team [7]. Equally, patients with nutritional impact symptoms should be reviewed by an appropriate specialist (e.g. supportive care team, palliative care team) [7].
  It should be noted that ESPEN define a nutrition support team as “a multi-disciplinary team of physicians, dietitians, nurses and pharmacists” (and other healthcare professionals), whose primary objective is “to support hospital staff in the provision of nutrition therapy, especially enteral or parenteral nutrition, to ensure that the nutritional needs of patients are satisfied, especially for those patients with complicated nutritional problems” [13].
3. Any decision to initiate clinically assisted nutrition should be made by an appropriately constituted multidisciplinary healthcare team together with the patient and their family [Level of evidence—V; category of guideline—suggestion].
  The decision to initiate (or not) CAN/other nutritional therapies depends on a number of factors (Table 3), and so requires input from the oncology team, the specialist dietitian/nutrition support team, the supportive care/palliative care team, and the patient and their family. Patients with rapidly progressive disease, patients with evidence of significant systemic inflammation (i.e. increased C-reactive protein with decreased albumin), and patients with a poor performance status (i.e. Eastern Cooperative Oncology Group performance status ≥ 3) are less likely to derive benefit from CAN [7]. However, the decision remains somewhat subjective due to the limited evidence in this cohort of patients [10, 36], and the difficulty/complexity of prognostication in this cohort of patients [17].
  The “stable” Cochrane systematic review of medically assisted nutrition for adult palliative care patients (i.e. “patients receiving palliative care”) [10] identified four prospective uncontrolled studies involving cancer patients [37–40], but no randomised controlled trials. The authors of this systematic review concluded that “There are insufficient good-quality studies to make any recommendations for practice with regards to the use of medically assisted nutrition in palliative care patients” [10]. It should be noted that this systematic review included studies involving patients with cancer and patients with other life limiting conditions.
  A recent systematic review of parenteral nutrition in patients with advanced cancer (i.e. “not curable but might respond to cancer treatment or disease-directed therapy to prolong life and reduce symptoms”) [36] identified two randomised controlled trials [41, 42], five prospective uncontrolled studies [43–47], and one retrospective uncontrolled study [48]. The authors of this systematic review concluded that “Current PN treatment in patients with advanced cancer is understudied and the level of evidence is weak” [36]: the authors further concluded that “Regardless of anti-neoplastic treatment and GI function, nutritional status seems to be improved by current PN treatment in malnourished patients. No benefit on survival of PN in terminal patients or patients able to feed enterally were reported. The frequency of adverse effects was low; however, a lack of systematic reporting was observed”. It appears that there is no analogous systematic review of enteral tube feeding in patients with advanced cancer.
  Since this systematic review was published, further studies on parenteral nutrition in advanced cancer have been reported [49, 50]. Thus, Bouleuc et al. (2020) reported a randomised controlled trial of parenteral nutrition versus oral feeding in patients with advanced cancer and malnutrition (and a functioning gastrointestinal tract) [49]: in this cohort of patients, parenteral nutrition was not associated with improved health related quality of life, or survival, but was associated with more adverse effects. Similarly, Amona et al. (2020) reported a secondary analysis of a prospective observational study of end-of-life care in palliative care units in Japan [50]: in this cohort of patients, enteral and parenteral nutrition was associated with increased survival as compared to oral feeding.
4. Clinically assisted nutrition should be considered in patients with an inability (reversible/irreversible) to ingest sufficient nutrients [Level of evidence—V; category of guideline—suggestion].
  In some patients with advanced cancer, the cause of the nutritional disturbance is the inability to ingest sufficient food due to problems relating to the cancer and/or the cancer treatment, e.g. dysphagia from an oesophageal carcinoma. The underlying cause may or may not be reversible, and so CAN may be required in the short term or indefinitely (and may be required to either supplement or replace usual oral intake). For instance, a common application of enteral feeding is to support patients with oral mucositis during/following head and neck (chemo-) radiotherapy [7]. Importantly, irrespective of the clinical situation, the generic principles around decision-making about CAN still apply.
5. Clinically assisted nutrition should be considered in patients with an inability (reversible/irreversible) to absorb sufficient nutrients [Level of evidence—V; category of guideline—suggestion].
  In other patients with advanced cancer, the cause of the nutritional disturbance is the inability to digest sufficient food due to problems relating to the cancer and/or the cancer treatment, e.g. surgical resection of small bowel. The underlying cause may or may not be reversible, and so CAN may be required in the short term or indefinitely (and may be required to either supplement or replace usual oral intake). A common application of parenteral feeding is to support patients with malignant bowel obstruction secondary to gastrointestinal or gynaecological malignancies [51]. Many patients with malignant bowel obstruction have issues with both the ingestion of food, and the digestion of food. Importantly, irrespective of the clinical situation, the generic principles around decision-making about CAN still apply .
6. Clinically assisted nutrition should be considered in patients at risk of dying from malnutrition before dying from their cancer [Level of evidence—V; category of guideline—suggestion].
  One of the main indications for CAN in this cohort of patients is the prevention of premature death from malnutrition (as opposed to inevitable death from the cancer) [8: *Druml *et al.,* 2016*]. As discussed, the data indicates that young healthy adult males with no intake will starve to death in ~ 2 months [22], and this time period is expected to be “considerably reduced” in patients with cancer [24]. Thus, our suggestion is that relevant cancer patients with an estimated prognosis of > 1 month should be considered for CAN, but that cancer patients with a prognosis of days to short weeks should generally not be considered for CAN (unless there is another indication — see below).
  Moreover, our suggestion is that in cases of uncertainty (of prognosis), a trial of CAN should be considered (with precise criteria for continuation/discontinuation) [8]. It should be noted that guidelines on the use of parenteral nutrition differ somewhat in terms of “cut-offs” for expected prognosis (i.e. 1–3 months) [7]. The other potential indications for CAN in this cohort of patients are management of hunger (and thirst), and “preserving” of quality of life [8]. However, it is unclear what the specific criteria are for the latter indication.
7. Clinically assisted nutrition is not indicated for the treatment of cancer cachexia [Level of evidence—V; category of guideline—suggestion].
  Cancer cachexia is defined as “a multifactorial syndrome characterised by an ongoing loss of skeletal muscle mass (with or without loss of fat mass) that cannot be fully reversed by conventional nutritional support and leads to progressive functional impairment” [31]. Indeed, CAN is not indicated/recommended for the treatment of cancer cachexia [28], although oral nutritional supplements may be useful as part of a multimodal intervention [52].
8. Protocols/processes should be in place to deal with conflicts over the initiation (or withdrawal) of clinically assisted nutrition [Level of evidence—V; category of guideline—suggestion].
  The provision of CAN is often an emotive subject for patients and their families (particularly at the end-of-life) [2, 53]. As discussed, CAN is a medical treatment, and patients (and/or their families) do not have the right to demand the treatment. In cases of conflict, it is recommended obtaining a second opinion from a suitably qualified healthcare professional: other options such as involvement of a clinical ethics committee, or involvement of the legal system are not generally required in this cohort of patients [8].
9. Patients receiving clinically assisted nutrition should have a nutritional care plan which defines the agreed objectives of treatment, and the agreed conditions for withdrawal of treatment [Level of evidence—V; category of guideline—suggestion].
  Patients receiving CAN should have a nutritional care plan which includes the rationale for treatment, the specifics of treatment (e.g. method of CAN), details about ongoing follow-up, details about ongoing reassessment, the indications for continuation of treatment, the indications for discontinuation of treatment, and contact details for the specialist dietitian/nutritional support team (and other relevant healthcare professionals) [7, 8].
10. Enteral tube feeding is generally preferable to parenteral nutrition (if possible) [Level of evidence—I; category of guideline—recommendation].
  Expert opinion is that the enteral route should be used in preference to the parenteral route, with the parenteral route being used in cases where enteral tube feeding is either inadequate, or inappropriate (or impossible) [7]. The rationale involves lower adverse effects, ease of usage, and lower direct costs (and similar effectiveness) [7]. In terms of adverse effects, a recent meta-analysis determined that enteral tube feeding is associated with fewer infectious complications (e.g. wound infection, pneumonia), but similar levels of non-infectious complications (e.g. nausea and vomiting, diarrhoea), as compared to parenteral nutrition [54].
11. Clinically assisted nutrition should be available in all settings, including the home setting [Level of evidence—IV; category of guideline—suggestion].
  The provision of CAN for patients with advanced cancer is feasible (and safe) in the home/similar settings [37, 39, 40, 42–45, 47, 48, 51, 55], and so a planned discharge from hospital should not be a major factor in the decision to withhold/withdraw relevant treatments. Recently, ESPEN produced detailed guidance on the provision of enteral nutrition at home [56], and also on the provision of parenteral nutrition at home [57].
12. All patients receiving clinically assisted nutrition should be regularly reassessed [Level of evidence—V; category of guideline—suggestion].

**Table.2:** Recommendations/suggestions on clinically assisted nutrition in patients with advanced cancer

1 - All patients with advanced cancer should have regular nutritional assessments [Level of evidence - V; category of guideline - suggestion].
2 - Patients with nutritional problems should be reviewed by a specialist dietitian (with / without other members of the nutrition support team) [Level of evidence - V; category of guideline - suggestion].
3 - Any decision to initiate clinically assisted nutrition should be made by an appropriately constituted multidisciplinary healthcare team together with the patient and their family [Level of evidence - V; category of guideline - suggestion].
4 - Clinically assisted nutrition should be considered in patients with an inability (reversible / irreversible) to ingest sufficient nutrients [Level of evidence - V; category of guideline - suggestion].
5 - Clinically assisted nutrition should be considered in patients with an inability (reversible / irreversible) to absorb sufficient nutrients [Level of evidence - V; category of guideline - suggestion].
6 - Clinically assisted nutrition should be considered in patients at risk of dying from malnutrition before dying from their cancer [Level of evidence - V; category of guideline - suggestion].
7 - Clinically assisted nutrition is not indicated for the treatment of cancer cachexia [Level of evidence - V; category of guideline - suggestion].
8 - Protocols / processes should be in place to deal with conflicts over the initiation (or withdrawal) of clinically assisted nutrition [Level of evidence - V; category of guideline - suggestion].
9 - Patients receiving clinically assisted nutrition should have a nutritional care plan which defines the agreed objectives of treatment, and the agreed conditions for withdrawal of treatment [Level of evidence - V; category of guideline - suggestion].
10 - Enteral tube feeding is generally preferable to parenteral nutrition (if possible) [Level of evidence - I; category of guideline - recommendation].
11 - Clinically assisted nutrition should be available in all settings, including the home setting [Level of evidence - IV; category of guideline - suggestion].
12- All patients receiving clinically assisted nutrition should be regularly reassessed [Level of evidence - V; category of guideline - suggestion].

**Table.3:** Factors influencing the decision to initiate clinically assisted nutrition in patients with advanced cancer

Estimated prognosis*
Current nutritional status
Oral intake
Nutritional impact symptoms
Systemic inflammation
Cancer stage / trajectory
Options for further anticancer treatment
Performance status
Co-morbidities
Patient preference
Gastrointestinal tract functioning
Logistics (of providing clinically assisted nutrition)

* Prognosis is dependent on many of the other factors

All patients receiving CAN should be regularly reassessed with regard to the continuation, amendment, or discontinuation of the relevant treatment [8]. The objectives of reassessment are to (a) ensure the CAN is meeting the patient’s nutritional requirements; (b) ensure the CAN is well tolerated; (c) ensure the CAN remains acceptable (to the patient); and (d) ensure the CAN remains appropriate/consistent with the “goals of care”. Patients receiving TPN require regular biochemical monitoring, whilst patients receiving enteral tube feeding require minimal biochemical monitoring [56, 57].

A decision to withdraw CAN is not a decision to stop feeding, and relevant patients require a new nutritional care plan (which often involves so-called comfort feeding) [8]. Importantly, many patients in the terminal phase do not experience hunger, and those patients in the terminal phase that do experience hunger appear to respond to “small amounts” of food [58].

## Conclusion

CAN is a well-established medical intervention, which is primarily indicated for the prevention of death from malnutrition in selected individuals from specific groups of patients with advanced cancer, i.e. patients with an inability to ingest sufficient nutrients, and/or an inability to digest sufficient nutrients. CAN is not indicated for the management of anorexia, weight loss, cancer cachexia, or reduced oral intake due to nutrition impact symptoms (generally).

## Supplementary Information

Below is the link to the electronic supplementary material.

Supplementary file1 (DOCX 14 KB)

Supplementary file2 (DOCX 14 KB)

## Data Availability

Not applicable.
