# Supplementary material for: Multinational Association of Supportive Care in Cancer (MASCC) expert opinion/guidance on the use of clinically assisted nutrition in patients with advanced cancer
Source: Support Care Cancer. 2021 Oct 19;30(4):2983–92. doi: 10.1007/s00520-021-06613-y (PMC8857106; doi:10.1007/s00520-021-06613-y)
Supplement: Supplementary file 1 — Supplementary file1 (DOCX 14 KB) [file 520_2021_6613_MOESM1_ESM.docx]

MULTINATIONAL ASSOCIATION OF SUPPORTIVE CARE IN CANCER (MASCC) EXPERT OPINION / GUIDANCE ON THE USE OF CLINICALLY ASSISTED NUTRITION IN PATIENTS WITH ADVANCED CANCER

Alderman B, Allan L, Amano K, Bouleuc C, Davis M, Lister-Flynn S, Mukhopadhyay S, Davies A (Prof Andrew Davies, Professor of Palliative Medicine, andavies@tcd.ie)

**APPENDIX 1 – MEDLINE search strategy**

1. Clinically assisted nutrition (keyword)

***or 2-11***

1. Medically assisted nutrition (keyword)
2. Artificial feeding (keyword)
3. Nutritional support (MESH term)
4. Enteral nutrition (MESH term)
5. Parenteral nutrition (MESH term)
6. Parenteral nutrition, total (MESH term)
7. Feeding methods (MESH term)
8. Nasogastric feeding (keyword)
9. Nasojejunal feeding (keyword)
10. Intubation, gastrointestinal (MESH term)

***and***

1. Cancer (keyword)

***or 13***

1. Neoplasms (MESH term)

***Limited to English language, human, all adult (19 plus years)***
