# Supplementary material for: Multinational Association of Supportive Care in Cancer (MASCC) expert opinion/guidance on the use of clinically assisted nutrition in patients with advanced cancer
Source: Support Care Cancer. 2021 Oct 19;30(4):2983–92. doi: 10.1007/s00520-021-06613-y (PMC8857106; doi:10.1007/s00520-021-06613-y)
Supplement: Supplementary file 2 — Supplementary file2 (DOCX 14 KB) [file 520_2021_6613_MOESM2_ESM.docx]

MULTINATIONAL ASSOCIATION OF SUPPORTIVE CARE IN CANCER (MASCC) EXPERT OPINION / GUIDANCE ON THE USE OF CLINICALLY ASSISTED NUTRITION IN PATIENTS WITH ADVANCED CANCER

Alderman B, Allan L, Amano K, Bouleuc C, Davis M, Lister-Flynn S, Mukhopadhyay S, Davies A (Prof Andrew Davies, Professor of Palliative Medicine, andavies@tcd.ie)

**APPENDIX 2 – MASCC criteria for grading recommendations [MASCC, 2020]**

Levels of evidence

| I | Evidence obtained from meta-analysis of multiple, well-designed, controlled studies; randomized trials with low false-positive and false-negative errors (high power) |
| --- | --- |
| II | Evidence obtained from at least one-well designed experimental study; randomized trials with high false-positive and/or false-negative errors (low power) |
| III | Evidence obtained from well-designed, quasi-experimental studies, such as nonrandomized, controlled single-group, pretest-posttest comparison, cohort, time, or matched case-control series |
| IV | Evidence obtained from well-designed, non-experimental studies, such as comparative and correlational descriptive and case studies |
| V | Evidence obtained from case reports and clinical examples |

Categories of guidelines

| Recommendation | Reserved for guidelines that are based on Level I or Level II evidence |
| --- | --- |
| Suggestion | Used for guidelines that are based on Level III, Level IV, and Level V evidence; this implies panel consensus on the interpretation of this evidence |
| No guideline possible | Used when there is insufficient evidence on which to base a guideline; this implies (1) that there is little or no evidence regarding the practice in question, or (2) that the panel lacks consensus on the interpretation of existing evidence |
